# The geometric accuracy of off‐axis targets in stereotactic body radiotherapy treatments across three linear accelerators

**DOI:** 10.1002/acm2.70383

**Published:** 2026-02-12

**Authors:** Dinesan Chinnaiya, Gopinath Mudhana

**Affiliations:** ^1^ Department of Physics School of Advanced Sciences Vellore Institute of Technology Chennai India; ^2^ Department of Radiation Oncology Sri Shankara Cancer Hospital & Research Centre Bangalore India

**Keywords:** geometric accuracy, SIMT–SBRT, Winston–Lutz test

## Abstract

**Purpose:**

This study quantifies isocenter and off‐axis geometric uncertainties using MultiMet Winston–Lutz (WL) tests to optimize gross tumour volume (GTV)‐to‐PTV (planning target volume) margin for single isocenter multiple target (SIMT) stereotactic body radiotherapy (SBRT) across three linear accelerators.

**Methods and Materials:**

Geometric inaccuracies were quantified for Trilogy (HD‐MLC, 6 MV SRS), TrueBeam (Millennium MLC), and TrueBeam STx (HD‐MLC, 6‐degrees‐of‐freedom (6DoF) couch) using a Sun Nuclear MultiMet‐WL cube containing six tungsten carbide markers arranged along the superior–inferior axis. The electronic portal imaging device (EPID) images acquired at four cardinal gantry angles with varied collimator/couch rotations were analyzed using MultiMet‐WL software (v2.1) to measure 3D (Δ) displacements for all the LINACs. The required GTV‐to‐PTV margins were calculated using a modified van Herk formula (2.5Σ+1.64σ), incorporating measured 3D displacements for isocenter and off‐axis targets.

**Results:**

The TrueBeam STx (HD‐MLC/6DoF) demonstrated superior geometric accuracy, maintaining ≤0.5 mm isocenter precision and ≤0.59 mm off‐axis targeting (3–7 cm). The Trilogy exceeded TG‐142 tolerances (1.06 ± 0.59 to 1.09 ± 0.57 mm) at all targets, requiring 4 mm uniform margins, while the TrueBeam (MMLC) showed optimal variations (isocenter: 0.68 ± 0.34 mm; superior off‐axis: 0.74 ± 0.36 mm). Both TrueBeam platforms achieved sub‐millimeter accuracy but demonstrated direction dependency for off‐axis targets, requiring 2–3 mm anisotropic margins. Notably, isotropic margins introduced up to 11% delineation errors for off‐axis targets due to these machine‐specific geometric variations, highlighting the imperative for platform‐specific margin protocols in SIMT SBRT.

**Conclusion:**

This study demonstrates that routine analysis of MultiMet WL testing is an essential tool for the spatial accuracy of the LINAC to establish machine‐specific PTV margin expansion in SIMT‐SBRT, particularly for targets where rotational errors dominate.

## 1 INTRODUCTION

Single isocenter multiple target (SIMT) stereotactic body radiotherapy (SBRT) has revolutionized the management of oligometastatic pulmonary disease, establishing itself as the standard of care for patients with 2–5 lesions due to its superior local control rates and favorable toxicity profile.^1^, ^2^, ^3^, ^4^, ^5^ While conventional multiple isocenter techniques have demonstrated clinical efficacy, they present significant practical limitations. Each lesion requires individual CBCT‐guided alignment, resulting in protracted treatment sessions that often exceed the tolerance thresholds of typically elderly or debilitated patients.^6^, ^7^, ^8^ These extended treatment times compromise patient comfort and increase the likelihood of intrafraction motion errors, potentially jeopardizing treatment accuracy.^1^ The advent of single‐isocenter multiple target stereotactic body radiotherapy (SIMT SBRT) using volumetric modulated arc therapy (VMAT) has addressed these challenges by enabling simultaneous treatment of multiple lesions through a single setup, dramatically improving treatment efficiency by reducing delivery time up to 50% while maintaining comparable plan quality to traditional approaches.^9^, ^10^

SIMT SBRT demands exceptional spatial accuracy to ensure precise dose delivery across multiple lesions while sparing surrounding healthy tissues. Achieving this level of accuracy requires a thorough understanding of the location of the isocenter in the linear accelerator and its impact on multiple target treatments.^11^ The isocenter, where all treatment beams converge, is the foundation for spatial precision in SIMT SBRT. Unlike single‐target SBRT, where errors primarily affect one lesion, misalignment in SIMT treatments can propagate across multiple targets. This may lead to geometric misses with underdosing of tumors due to positional errors, increased dose to organs‐at‐risk from misdirected high‐gradient beams, and dosimetric degradation caused by rotational uncertainties affecting off‐isocenter lesions. Thus, precise isocenter calibration and stability are critical for safe and effective SIMT SBRT delivery.^12^, ^13^

Several factors contribute to spatial inaccuracies in SIMT treatments. LINAC mechanical isocenter stability is paramount, as gantry, collimator, and couch rotations must align within sub‐millimeter tolerances of less than 1 mm (AAPM Task Group 101).^14^, ^15^ Wobble effects, caused by mechanical flex during gantry rotation, can displace the radiation beam relative to the intended isocenter.^16^ To ensure high spatial accuracy in SIMT SBRT, rigorous LINAC isocenter verification is essential. Winston–Lutz (WL) tests are routinely performed to quantify the coincidence between the radiation and mechanical isocenter. At the same time, high‐frequency quality assurance protocols help detect drifts in gantry and couch alignment.^15^, ^17^ Quality assurance for SIMT SBRT demands specialized approaches to ensure the precise delivery of ablative doses to small, multiple targets. End‐to‐end testing with multiple target phantoms verifies isocenter accuracy, MLC positioning, and target localization accuracy across all lesions.^18^

The clinical consequences of spatial errors in SIMT SBRT can be significant. Even sub‐millimeter deviations may result in underdosing of small lesions, compromising tumor control. Conversely, misalignment near critical structures risks overdosing sensitive organs, potentially leading to severe toxicities.^19^ To account for misaligned isocenter or geometrical inaccuracies, the planning target volume (PTV) margin is recommended in terms of PTV distance from the isocenter. Instead of an isotropic margin, the PTV margin is calculated toward the direction of the uncertainties.^20^ This study aims to quantify the variation of isocenter and off‐axis center misalignment in SIMT SBRT through comprehensive WL analysis and to establish a robust margin optimization framework that systematically addresses the geometric uncertainties inherent in off‐axis target irradiation. By correlating WL‐measured mechanical inaccuracies, this study proposes to derive machine‐specific PTV expansion criteria that account for both the magnitude and directionality of the errors, thereby advancing the precision of multi‐metastatic SBRT delivery.

## 2 MATERIALS AND METHODS

Our study employed three state‐of‐the‐art Varian linear accelerators (Varian Medical System, Palo Alto, CA) to evaluate off‐axis target center accuracy for SIMT SBRT comprehensively. The Trilogy CD2300 system features a high‐definition multi‐leaf collimator (HD‐MLC) (2.5 mm central leaves) specifically configured with a 6 MV stereotactic radiosurgery (SRS) beam capable of delivering 1000 MU/min for high‐dose treatments. The TrueBeam STx platform combines the precision of an HD‐MLC with advanced 6‐degrees‐of‐freedom (6DoF), enabling both effective beam modulation^21^, ^22^ and sub‐millimeter patient positioning corrections in all rotational directions. We included another TrueBeam system equipped with 120 Millennium MLC (MMLC) (5 mm central leaves). Both TrueBeam units have flattening filter‐free (FFF) beams with high dose rate capabilities to deliver conventional and SIMT SBRT.

**FIGURE 1:**
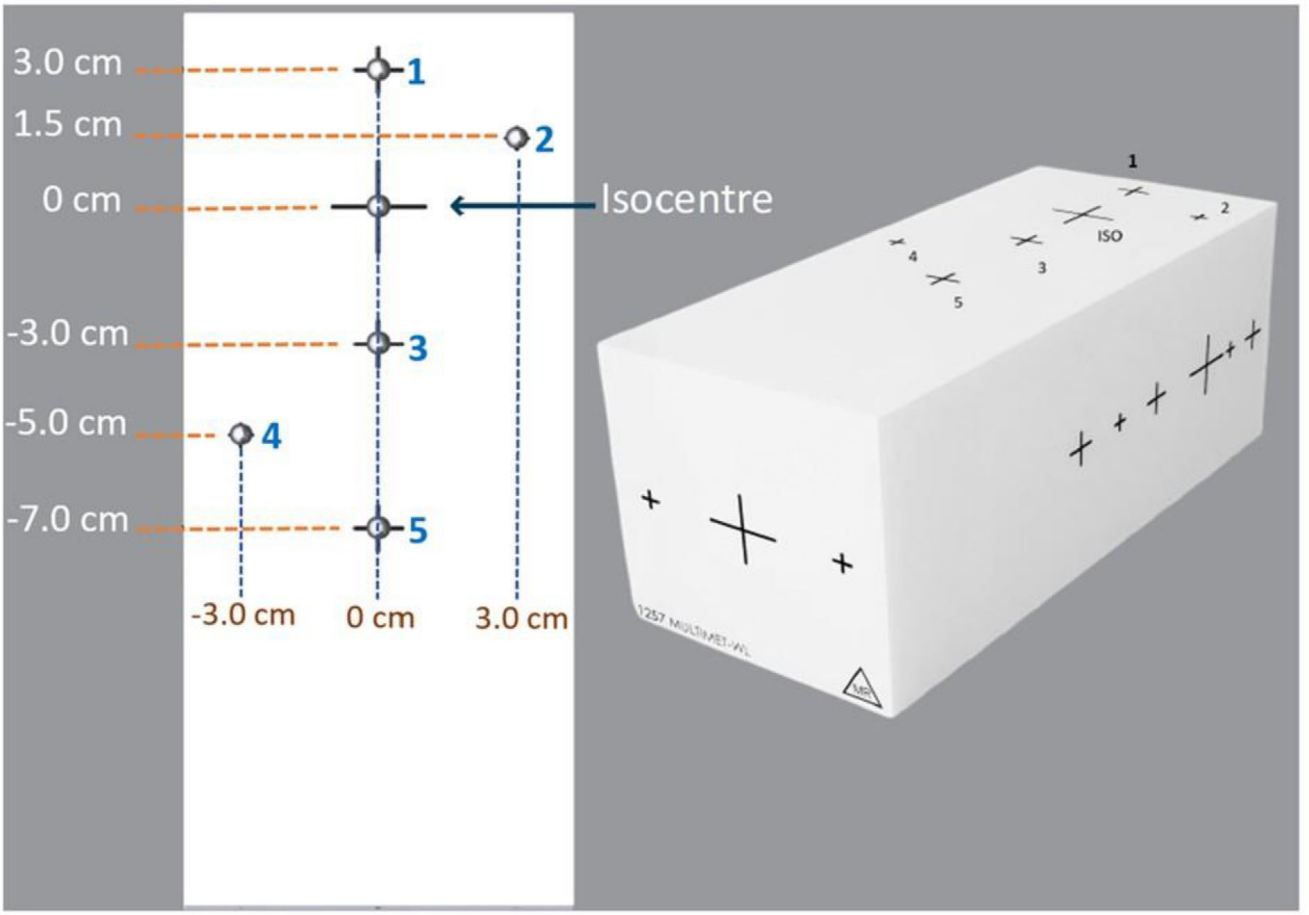
Schematic of MultiMet Winston–Lutz phantom (AP View) with off‐axis targets at defined distances (superior, inferior, and lateral axes).

**FIGURE 2:**
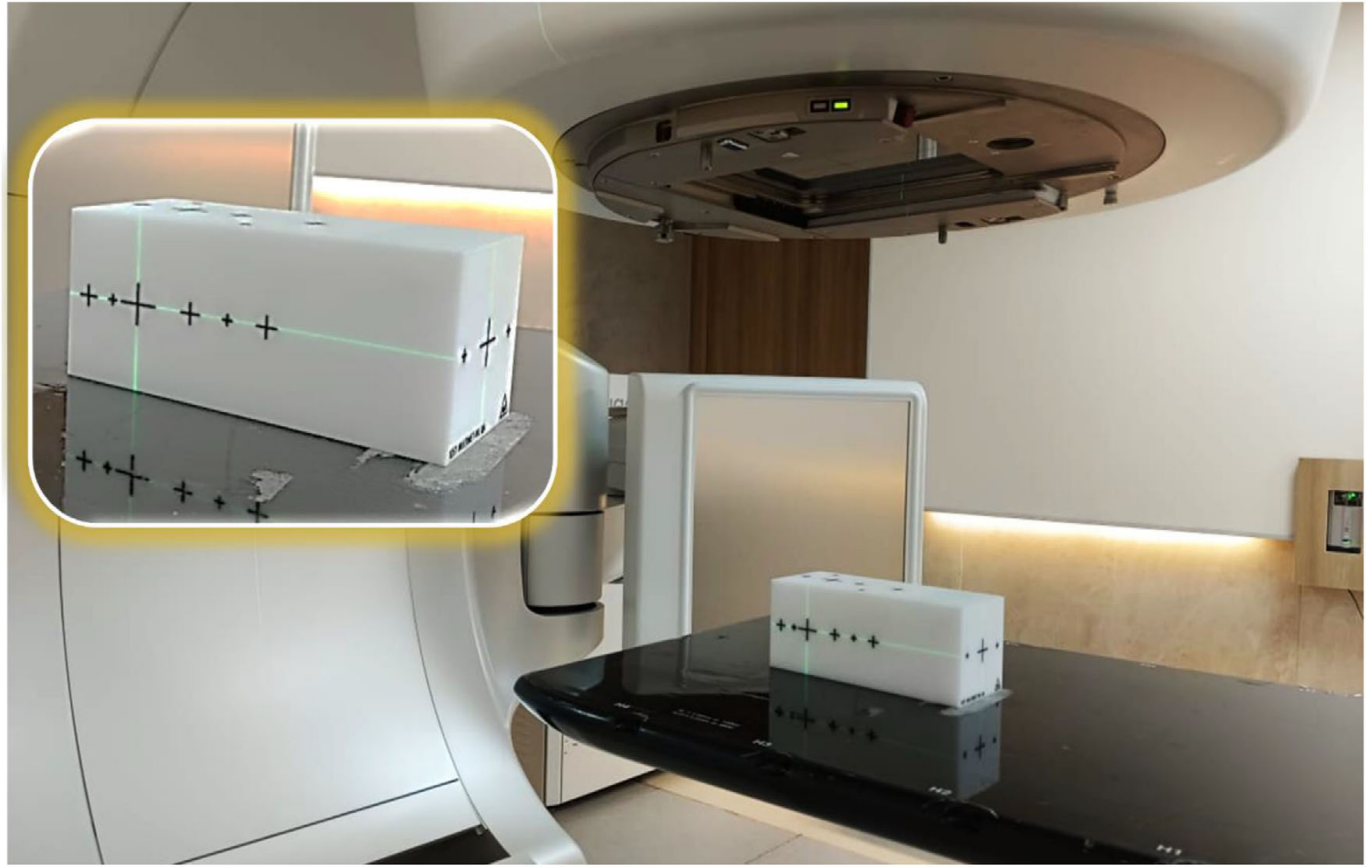
Winston–Lutz phantom setup on TrueBeam STx couch, with lasers aligned to phantom marks for isocenter QA.

**FIGURE 3:**
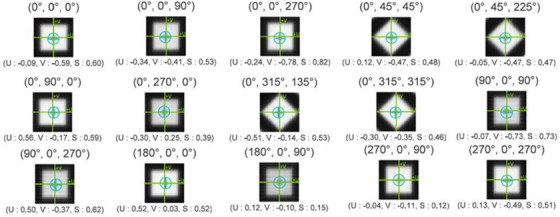
Processed DICOM images from the MultiMet‐WL software showing detected tungsten carbide marker positions (marked by blue circles with plus signs) and their measured offsets from the isocenter (green crosshair) for each beam geometry.

The geometric accuracy of off‐axis target position was evaluated using a specialized multiple target cube (StereoPHAN MultiMet‐WL, Sun Nuclear Corporation). The measurements were carried out over 2 months to rule out the consistency and reproducibility. This precision acrylic cube (85 × 85 × 127.5 mm) contains six tungsten carbide (5.00 mm diameter ± 25 µm tolerance) arranged in a co‐planar configuration. The central carbide was designated as the primary isocenter reference point (origin [0,0,0]), with five additional carbides positioned at known off‐sets along the superior–inferior and lateral (targets 2 and 4) axes for off‐axis measurements (Figure 1). For the experimental setup, the cube was mounted on the treatment couch, and initial alignment was performed using in‐room lasers, followed by verification with kilovoltage cone‐beam CT imaging (Figure 2). In order to analyze the positional accuracy of different targets, image acquisition was performed using the electronic portal imaging device (EPID) at four cardinal gantry angles (0°, 90°, 180°, and 270°) with different collimator and couch rotations to include all geometric degrees of freedom (Table 1) across all three LINACs. The protocol includes 19 beams in total, with beams numbered 4, 6, 8, and 10 repeated to cover tungsten carbide inserts at targets 2 and 4, specifically at gantry angles 90° and 270°. The DICOM images acquired from the EPID were processed using the MultiMet‐WL Analysis tool (v2.1, Sun Nuclear Corporation, Melbourne, Florida, USA) to detect both the radiation field edges and the projected positions of the tungsten carbide markers shown in Figure 3. The centroids of each sphere's radiographic signature and the corresponding beam apertures were calculated with subpixel accuracy. For each gantry angle (*θ*) and couch angle (*φ*), the 2D EPID coordinates (*u*,*v*) were transformed to the patient reference frame using a vector transformation matrix (Equations 1 and 2).^23^ The 3D displacement vectors were then determined through a pseudo‐inverse calculation of the measured positions (Equation 3).^22^

(1) $$\begin{array}{cc} \left(\begin{array}{c} u\\ v \end{array}\right) & =\left(\begin{array}{ccc} \cos(\theta )\cos(\phi ) & -\cos(\theta )\sin(\phi ) & \sin(\theta )\\ \sin(\phi ) & \cos(\phi ) & 0 \end{array}\right)\\ & \;\times \left(\begin{array}{c} \Delta \mathrm{VERT}\\ \Delta \mathrm{LAT}\\ -\Delta \mathrm{AP} \end{array}\right) \end{array}$$

(2) $$\xi ={\left(\Delta v_{1},\Delta u_{1},\Delta v_{2},\Delta u_{2}\right)}^{T}$$

(3) $$\Delta ={\left(A^{T}A\right)}^{-1}A^{T}\xi$$

(4) $$\mathrm{Systematic}\;\mathrm{Error}=\sum =\frac{\sum_{i}^{N}\Delta i}{N}$$

(5) $$\mathrm{Random}\;\mathrm{Error}=\sigma =\sqrt{\frac{\sum_{i}^{N}{\left(\Delta_{i}-\sum \right)}^{2}}{\left(N-1\right)}}$$

**TABLE 1:** Beam geometries for Winston–Lutz testing, showing gantry, collimator, couch angles, and target coverage.

Beam #	Gantry	Collimator	Couch	Targets covered
1	180	0	0	All
2	180	90	0	All
3	90	90	0	Iso, 1, 3, 5
4	90	90	0	2, 4
5	90	270	0	Iso, 1, 3, 5
6	90	270	0	2, 4
7	270	270	0	Iso, 1, 3, 5
8	270	270	0	2, 4
9	270	90	0	Iso, 1, 3, 5
10	270	90	0	2, 4
11	0	90	0	All
12	0	0	0	All
13	0	270	0	All
14	0	0	270	All
15	0	45	315	All
16	0	225	315	All
17	0	135	45	All
18	0	315	45	All
19	0	0	90	All

*Note*: Only 15 unique beams were analyzed; repeated geometric parameters are counted as a single evaluation.

The PTV margin for off‐axis targets in SBRT must incorporate geometric uncertainties derived from 3D displacement measurements (Δ) of tungsten carbide markers acquired during Winston–Lutz testing (Equation 3). Using the coordinate transformation framework established in the previous section, these margins can be computed by combining both systematic (*Σ*) and random (*σ*) error components (Equations 4 and 5).^20^, ^24^ These error terms were determined from a comprehensive analysis of translational (lateral, longitudinal, and vertical) and rotational (pitch, roll, and yaw) setup variations quantified through WL measurements. The rotational deviations were converted to equivalent translational displacements using Sarkar et al.'s mathematical derivations.^25^

Following van Herk's methodology,^20^ geometric uncertainties were combined in quadrature (root‐sum‐square) to determine the systematic error component (*Σ*) for the 2.5*Σ* + 1.64*σ* margin expansion formula. PTV margins were then calculated for each tungsten carbide marker position across all three linear accelerator platforms.

## 3 RESULTS

### 3.1 Mechanical test

The couch and collimator positioning accuracy of all three linear accelerators were rigorously evaluated by the MultiMet‐WL cube, and the results were presented separately in Tables 2 and 3. Table 2 quantifies couch rotation accuracy, revealing maximum angular errors of 0.2° for the Trilogy, 0.1° for the TrueBeam STx, and 0.4° for the TrueBeam system at four cardinal angles (0°, 90°, 180°, and 270°). Table 3 shows the collimator rotation accuracy test results, demonstrating excellent positioning accuracy across all measured angles. The data indicate consistent performance within a tight ± 0.2° range of the expected values, with several positions achieving perfect alignment with no differences and others maintaining accuracy (0.0–0.1° difference). The maximum observed deviation was just 0.2°, confirming the collimator's ability to maintain high positional accuracy throughout its full rotational range across all the machines.

**TABLE 2:** Measured couch rotation accuracy across test angles when the gantry and collimator were set at 0° for all linear accelerator platforms.

	Trilogy		TrueBeam STx		TrueBeam	
Expected	Measured	Difference	Measured	Difference	Measured	Difference
45.0°	44.9°	0.1°	45.1°	0.1°	44.7°	0.3°
90.0°	89.8°	0.2°	90.0°	0.0°	89.6°	0.4°
270.0°	270.2°	0.2°	270.0°	0.0°	270.2°	0.2°
315.0°	315.1°	0.1°	315.0°	0.0°	315.1°	0.1°

**TABLE 3:** Measured collimator rotation accuracy across test angles when the gantry and couch were set at 0° for all linear accelerator platforms.

	Trilogy		TrueBeam STx		TrueBeam	
Expected	Measured	Difference	Measured	Difference	Measured	Difference
45.0°	45.2°	0.2°	45.0°	0.0°	44.8°	0.2°
90.0°	89.8°	0.2°	89.9°	0.1°	89.9°	0.1°
135.0°	135.1°	0.1°	134.8°	0.2°	134.9°	0.1°
225.0°	225.0°	0.0°	225.0°	0.0°	225.0°	0.0°
270.0°	270.1°	0.1°	270.0°	0.0°	270.0°	0.0°
315.0°	315.1°	0.1°	315.0°	0.0°	315.0°	0.0°

### 3.2 MultiMet WL test

**FIGURE 4:**
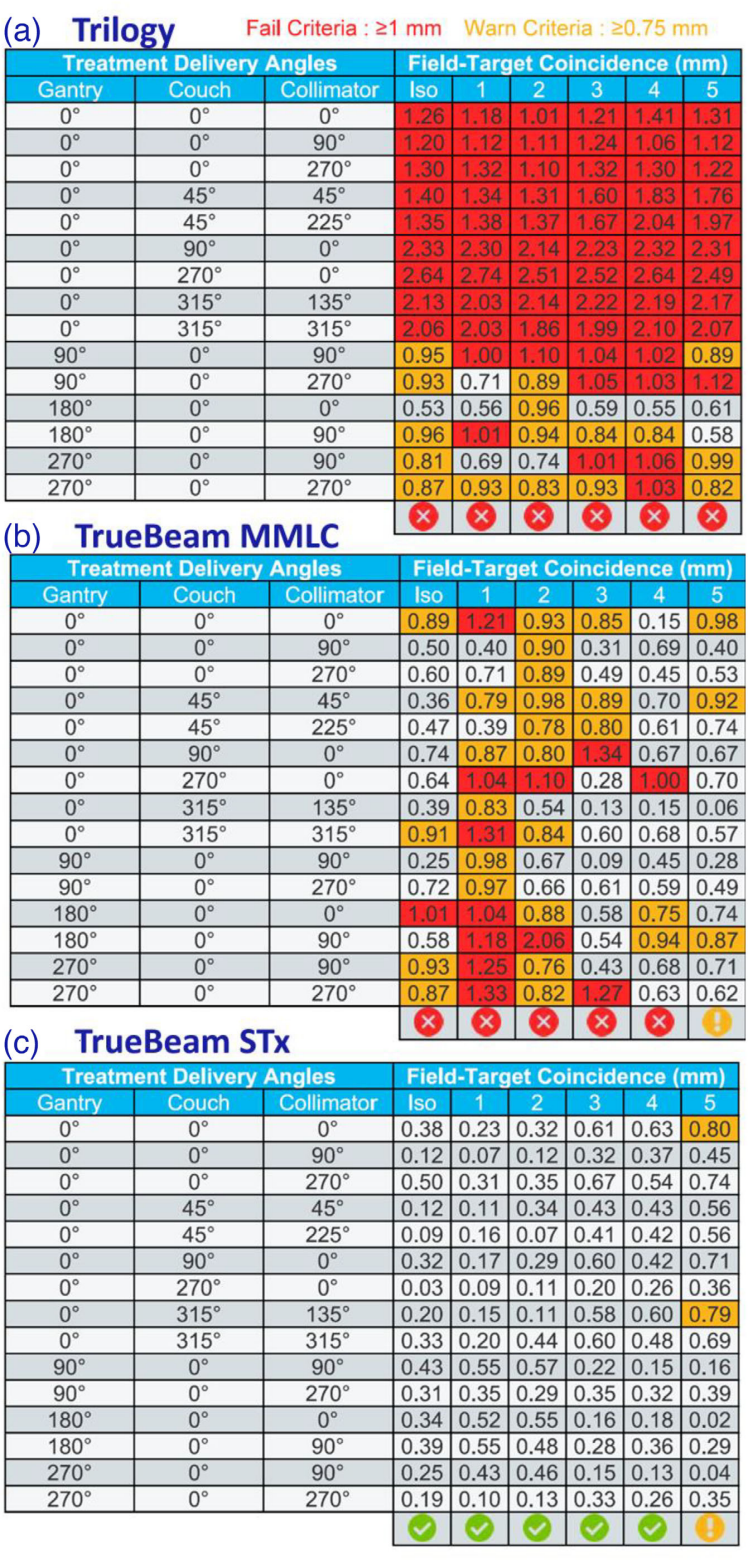
WL test performance comparison failure rates and warning occurrences in Trilogy, TrueBeam, and TrueBeam STx LINACs.

Figure 4 presents the MultiMet WL representative test results carried out on a single day for off‐axis targets across each LINAC platform, tabulated as a function of distance from the isocenter. The passing criterion for each target was an accuracy of <1 mm, while the warning threshold was set at ≥0.75 mm.^26^ Figure 4a presents the results of the Trilogy showing that most targets failed to meet acceptability criteria across all beam geometries on every measurement day. Figure 4b displays the WL test results for the TrueBeam with the MMLC system, showing frequent failures to meet specifications. In contrast, Figure 4c shows that none of the measurement points failed, and only a few points met the warning criteria for the TrueBeam STx LINAC.

Table 4 summarizes the mean of the geometric inaccuracies with SD for isocenter and off‐axis targets across three LINAC platforms carried out over the entire course of study. Trilogy (HD‐MLC) demonstrated consistently elevated deviations (1.06 ± 0.59 to 1.09 ± 0.57 mm) exceeding clinical tolerances, with uniform inaccuracies across all positions (Δ0.03 mm range) and moderate variability (±0.55 to ±0.60 mm). TrueBeam 120 MMLC (3DoF couch) maintained optimal performance at the isocenter (0.68 ± 0.34 mm) but showed progressive degradation at off‐axis positions (0.74 ± 0.36 mm to 0.76 ± 0.37 mm superior; 0.68 ± 0.34 to 0.72 ± 0.36 mm inferior targets). Most impressively, TrueBeam STx (HD‐MLC) with 6DoF couch achieved superior precision, with sub‐millimeter accuracy at isocenter (0.41 ± 0.19 mm) and 1.5 cm superior position (0.37 ± 0.24 mm). However, it exhibited significantly increased deviations at inferior positions (0.52 ± 0.23 to 0.59 ± 0.27 mm) (*p* < 0.05). All systems demonstrated consistent variability (±0.19–0.60 mm) across measurements, with the TrueBeam STx showing the highest positional accuracy overall.

**TABLE 4:** Geometric deviations (mean ± SD) in mm for three LINAC platforms, summarizing descriptive statistics of isocenter and off‐axis target positional accuracy over multiple days.

Carbide position (*x*, *y*, *z*) cm	Trilogy (HD‐MLC)	TrueBeam 120 MMLC	TrueBeam STx (HD‐MLC)
ISO (0, 0, 0)	1.06 ± 0.59	0.68 ± 0.34	0.41 ± 0.19
(0, 3.0, 0)	1.08 ± 0.60	0.74 ± 0.36	0.39 ± 0.24
(3.0, 1.5, 0)	1.09 ± 0.57	0.76 ± 0.37	0.37 ± 0.24
(0, −3.0, 0)	1.06 ± 0.55	0.68 ± 0.34	0.52 ± 0.23
(−3.0, −5.0, 0)	1.08 ± 0.59	0.69 ± 0.34	0.55 ± 0.23
(0, −7.0, 0)	1.08 ± 0.58	0.72 ± 0.36	0.59 ± 0.27

### 3.3 Geometric inaccuracies versus PTV margin

PTV margins must account for both systematic (*Σ*) and random (*σ*) errors to ensure treatment delivery accuracy. The target displacements measured in our Winston–Lutz tests (Table 4) quantify machine‐related geometric inaccuracies that should be incorporated into margin recipes (van Herk's 2.5*Σ* + 1.64*σ*)^20^ to maintain adequate tumor coverage.

Table 5 presents the minimum PTV margin requirements derived from measured geometric inaccuracies at isocenter and off‐axis positions for all three LINAC systems. Trilogy CD2300 demonstrated the largest margins, ranging from 3.84 mm (isocenter) to 3.89 mm (1.5 cm superior and 3 cm left lateral position), indicating consistently higher geometric inaccuracies. TrueBeam system required smaller margins, with 2.39 mm at the isocenter increasing to 2.49–2.67 mm for off‐axis targets, peaking at the 1.5 cm superior position (2.67 mm). Notably, TrueBeam STx achieved the tightest margins overall, maintaining 2.19 mm at isocenter and 2.14–2.43 mm for off‐axis targets, with the largest deviation (2.43 mm) occurring at 7 cm inferior.

**TABLE 5:** Comparative PTV margins in mm needed to compensate for geometric inaccuracies across LINAC platforms, measured at isocenter and off‐axis targets.

	PTV margin (mm)					
Targets	Trilogy	% Diff	TrueBeam 120 MMLC	% Diff	TrueBeam STx	% Diff
ISO	3.84		2.39		2.19	
3.0 cm/3.0 cm	3.87	0.9	2.63	5.5	2.17	−1.0
1.5 cm	3.89	1.4	2.67	**7.1**	2.14	−2.0
−3.0 cm	3.84	0.0	2.49	−0.2	2.33	6.5
−5.0 cm/−3.0 cm	3.87	0.9	2.49	−0.2	2.37	8.4
−7.0 cm	3.87	0.9	2.53	1.4	2.43	**11.1**

*Note*: The % Diff represents the percentage difference between the planning target volume (PTV) margins at the isocenter and those at the off‐axis targets.

## 4 DISCUSSION

The mechanical tests confirmed high positioning accuracy for couch and collimator rotations for all the linear accelerators, with maximum deviation of ≤0.4° for couch angles and ≤0.2° for collimator angles. This study utilized the Sun Nuclear MultiMet WL Cube to assess both isocentric and off‐axis positioning accuracy, covering a range of 3 cm superiorly and 7 cm inferiorly from the isocenter. The Trilogy CD2300 demonstrated systematic geometric inaccuracies, with deviations (1.06 ± 0.59 to 1.09 ± 0.57 mm) consistently exceeding clinical tolerances (<1 mm) (Task Group 142).^15^ The minimal positional variation (Δ0.03 mm range) and uniform variability (±0.55–0.60 mm), coupled with non‐significant superior–inferior differences (*p* > 0.05), suggest a systematic error rather than positional dependence affecting all directions equally. These findings have direct clinical relevance for SIMT SBRT lung treatments. The observed deviations necessitated a ≃4 mm Gross Tumour Volume/Internal Target Volume to PTV margin for all targets, irrespective of distance. This margin exceeds the Task Group‐101 recommendation^14^ potentially increasing dose to adjacent healthy tissues.

TrueBeam with MMLC LINAC demonstrated excellent isocentric accuracy (0.68 ± 0.34 mm) with degradation at off‐axis positions (0.74 ± 0.36 mm superiorly to 0.72 ± 0.36 mm inferiorly), all within clinical tolerances (<1 mm). This directional pattern suggests gravitational influence on the gantry or MMLC assembly, supported by the system's tight reproducibility (±0.34 to 0.37 mm). For SIMT SBRT lung treatments, these findings support a risk‐adapted margin strategy: ≃2 mm for central targets (aligning with TG‐101^14^) and 2–3 mm for off‐axis lesions, representing a 7% increase in PTV margin requirements for superior targets compared to isotropic delineation (Table 5). TrueBeam STx (HD‐MLC) demonstrates superior performance compared to other LINACs, achieving accuracy of ≤0.5 mm, which refers to the average displacement of all targets, including both isocenter and off‐axis positions. Although comparing individual targets exhibited deviations up to 0.59 mm at off‐axis, the mean displacement remained below 0.5 mm. Our calculated margins align with the recommended target margins for SIMT SBRT.^14^ However, when comparing isocenter versus off‐axis positions, we observed a 6%–11% increase in PTV margins for targets located 3–7 cm inferiorly (Table 5). For SIMT SBRT, field sizes typically range from 7 to 10 cm to encompass multiple lesions, consistent with the optimal PTV volume range (2–310 cc) reported for lung SBRT by Hoffman et al.^27^ In such cases, uniform PTV margins introduce up to 11% delineation error due to machine‐specific positional inaccuracies, particularly for off‐axis targets. These findings support implementing a risk‐adapted margin strategy in clinical practice, where PTV expansions are optimized based on both target location and machine‐specific performance characteristics. This approach is particularly crucial as our data reveal that each LINAC exhibits unique patterns of inaccuracies, while some systems show uniform deviations across all target positions, others demonstrate position‐specific variations (superior or inferior). The identical trend was not observed in this study—Trilogy and TrueBeam MMLC showed superior results, while TrueBeam STx exhibited an opposite pattern. This is because each machine's unique mechanical design, beam steering configurations, and calibration protocols lead to distinct isocenter behaviors. As Gao et al. reported^26^ such directional inaccuracies are machine‐specific, reinforcing the need for individual institutions to verify their LINAC's geometric stability through comprehensive QA. The observed variations are greater with Trilogy as it is very old (10 years) compared to TrueBeam STx, whereas TrueBeam MMLC is older only by 7 years, which results in less variation. This supports the understanding that newer systems tend to maintain tighter mechanical and dosimetric tolerances, resulting in better overall accuracy.

## 5 CONCLUSION

This study underscores the critical importance of machine‐specific geometric accuracy verification for accurate delivery of SIMT SBRT, particularly in the lung oligometastatic tumors. The uniform PTV margins advocate for a risk‐adapted approach, where margin expansions are tailored according to each LINAC's geometric behavior and the spatial positioning of targets. Such individualized strategies are essential to maintain optimal target coverage while minimizing exposure to surrounding healthy tissue. This study highlights the necessity of rigorous QA with MultiMet Winston–Lutz test to detect systematic and directional deviations in LINACs. Integrating these assessments into clinical protocols can enhance the tumor coverage and accuracy of SIMT SBRT treatment delivery across diverse clinical settings.

## AUTHOR CONTRIBUTIONS

Dinesan Chinnaiya is responsible for conceiving and designing the study, collecting and analyzing data, writing the manuscript, conducting a literature review, revising the manuscript, and managing the submission process. Gopinath Mudhana provided comprehensive project supervision, offered mentorship to the first author, assisted in refining research concepts, and contributed to manuscript review and approval.

## CONFLICT OF INTEREST STATEMENT

The author declares no conflict of interest.

## ETHICS APPROVAL

No ethics approval is required as the study does not involve humans or animals.

## Data Availability

The data that support the findings of this study are available from the corresponding author upon reasonable request.

## References

[1] Pokhrel D , Sanford L , Larkin S , et al. On the use of single‐isocenter VMAT plans for SBRT treatment of synchronous multiple lung lesions: plan quality, treatment efficiency, and early clinical outcomes. J Appl Clin Med Phys. 2020;21(8):160‐167. doi: 10.1002/acm2.12938 32432405

[2] Van Timmeren JE , Ehrbar S , Chamberlain M , et al. Single‐isocenter versus multiple‐isocenters for multiple lung metastases: evaluation of lung dose. Radiother Oncol. 2022;166:189‐194. doi: 10.1016/j.radonc.2021.11.030 34864135 10.1016/j.radonc.2021.11.030

[3] Katano A , Minamitani M , Ohira S , Yamashita H . Recent advances and challenges in stereotactic body radiotherapy. Technol Cancer Res Treat. 2024 ;23:15330338241229363. doi: 10.1177/15330338241229363 38321892 10.1177/15330338241229363 PMC10851756

[4] Chmura S , Winter K , Salama J , et al. Phase I trial of stereotactic body radiation therapy (SBRT) to multiple metastatic sites: an NRG oncology study. Int J Radiat Oncol Biol Phys. 2018;102:S68‐S69. doi: 10.1016/j.ijrobp.2018.06.187

[5] Sanford L , Molloy J , Kumar S , et al. Evaluation of plan quality and treatment efficiency for single‐isocenter/two‐lesion lung stereotactic body radiation therapy. J Appl Clin Med Phys. 2019;20(1):117‐127. doi: 10.1002/acm2.12500 10.1002/acm2.12500 PMC6333146 30548205

[6] Li Q , Mu J , Gu W , et al. Frameless stereotactic body radiation therapy for multiple lung metastases. J Appl Clin Med Phys. 2014;15(4):105‐115. doi: 10.1120/jacmp.v15i4.4737 10.1120/jacmp.v15i4.4737 PMC5875519 25207400

[7] Trager M , Salama J , Yin FF , et al. SBRT treatment of multiple extracranial oligometastases using a single isocenter with distinct optimizations. J Radiosurg SBRT. 2017;4:265‐273. 29296451 PMC5658822

[8] Graulieres E , Kubler S , Martin E , Ferrand R . Positioning accuracy of a single‐isocenter multiple targets SRS treatment: a comparison between Varian TrueBeam CBCT and Brainlab ExacTrac. Phys Med. 2020;80:267‐273. doi: 10.1016/j.ejmp.2020.10.022 33221708 10.1016/j.ejmp.2020.10.022

[9] O.M. Oderinde , Y. Voronenko , S. Tian et al. Dosimetric comparison of single‐isocenter and multiple‐isocenter techniques for two‐lesion lung SBRT using the RefleXion high‐speed ring‐gantry system. Int J Radiat Oncol Biol Phys. 2021;111(3):e139‐e140. doi: 10.1016/j.ijrobp.2021.07.582

[10] Raranje C , Mazur TR , Mo A , Laugeman E. Single‐isocenter, multiple‐target abdominal cone‐beam computed tomography (CBCT)‐guided online adaptive stereotactic body radiotherapy (SBRT). Cureus. 2024;16(9):e68904. doi: 10.7759/cureus.68904 39381481 10.7759/cureus.68904 PMC11458792

[11] Kido T , Ono T , Nakamura M , et al. Development and multi‐institutional evaluation of a new phantom for verifying beam‐positioning errors at off‐isocenter positions. Phys Med. 2023;112:102645. doi: 10.1016/j.ejmp.2023.102645 37478576 10.1016/j.ejmp.2023.102645

[12] Kim C , Kim H , Jung D , et al. Evaluation of the deliverability of dynamic conformal arc therapy (DCAT) by gantry wobble and its influence on dose. Sci Rep. 2024;14:7134. doi: 10.1038/s41598‐024‐57644‐4 38532018 10.1038/s41598-024-57644-4 PMC10965989

[13] Wu Q , Snyder K , Liu C , et al. Optimization of treatment geometry to reduce normal brain dose in radiosurgery of multiple brain metastases with single–isocenter volumetric modulated arc therapy. Sci Rep. 2016;6;34511. doi: 10.1038/srep34511 27688047 10.1038/srep34511 PMC5043272

[14] Benedict SH , Yenice KM , Followill D , et al. Stereotactic body radiation therapy: the report of AAPM Task Group 101. Med Phys. 2010;37(8):4078‐4101. doi: 10.1118/1.3438081 20879569 10.1118/1.3438081

[15] Klein EE , Hanley J , Bayouth J , et al. Task Group 142 report: quality assurance of medical accelerators. Med Phys. 2009;36(9):4197‐4212. doi: 10.1118/1.3190392 19810494 10.1118/1.3190392

[16] Du W , Johnson JL , Jiang W , Kudchadker RJ . On the selection of gantry and collimator angles for isocenter localization using Winston–Lutz tests. J Appl Clin Med Phys. 2016;17(1):167‐178. doi: 10.1120/jacmp.v17i1.5792 26894350 10.1120/jacmp.v17i1.5792 PMC5690203

[17] Hanley J , Dresser S , Simon W , et al. AAPM Task Group 198 Report: an implementation guide for TG 142 quality assurance of medical accelerators. Med Phys. 2021;48(10):e830‐e885. doi: 10.1002/mp.14992 34036590 10.1002/mp.14992

[18] Gao J , Liu X . Off‐isocenter Winston–Lutz test for stereotactic radiosurgery/stereotactic body radiotherapy. Int J Radiat Oncol Biol Phys. 2016;5:154‐161. doi: 10.4236/ijmpcero.2016.52017

[19] Nakano H , Tanabe S , Yamada T , et al. Maximum distance in single‐isocenter technique of stereotactic radiosurgery with rotational error using margin‐based analysis. Radiol Phys Technol. 2021;14:57‐63. doi: 10.1007/s12194‐020‐00602‐2 33393057 10.1007/s12194-020-00602-2

[20] van Herk M , Remeijer P , Rasch C , Lebesque JV . The probability of correct target dosage: dose‐population histograms for deriving treatment margins in radiotherapy. Int J Radiat Oncol Biol Phys. 2000;47(4):1121‐1135. doi: 10.1016/s0360‐3016(00)00518‐6 10863086 10.1016/s0360-3016(00)00518-6

[21] Petroccia HM , Malajovich I , Barsky AR , et al. Spine SBRT with Halcyon™: plan quality, modulation complexity, delivery accuracy, and speed. Front Oncol. 2019;9:319. doi: 10.3389/fonc.2019.00319 31106151 10.3389/fonc.2019.00319 PMC6498946

[22] Younge KC , Kuchta JR , Mikell JK , Rosen B , Bredfeldt JS , Matuszak MM . The impact of a high‐definition multileaf collimator for spine SBRT. J Appl Clin Med Phys. 2017;18(6):97‐103. doi: 10.1002/acm2.12197 10.1002/acm2.12197 PMC5689933 28960753

[23] Low DA , Li Z , Drzymala RE . Minimization of target positioning error in accelerator‐based radiosurgery. Med Phys. 1995;22(4):443‐448. doi: 10.1118/1.597475 7609726 10.1118/1.597475

[24] Prisciandaro JI , Frechette CM , Herman MG , Brown PD , Garces YI , Foote RL . A methodology to determine margins by EPID measurements of patient setup variation and motion as applied to immobilization devices. Med Phys. 2004;31(11):2978‐2988. doi: 10.1118/1.1800712 15587650 10.1118/1.1800712

[25] Sarkar B , Ray J , Ganesh T et al. Methodology to reduce 6D patient positional shifts into a 3D linear shift and its verification in frameless stereotactic radiotherapy. Phys Med Biol 2018;63(7):075004. doi: 10.1088/1361‐6560/aab231 29480166 10.1088/1361-6560/aab231

[26] Gao J , Anand D . Off‐iso Winston–Lutz test on seven linear accelerators. J Appl Clin Med Phys. 2024;25(10):e14470. doi: 10.1002/acm2.14470 39042435 10.1002/acm2.14470 PMC11466459

[27] Hoffman D , Dragojević I , Hoisak J , et al. Lung stereotactic body radiation therapy (SBRT) dose gradient and PTV volume: a retrospective multi‐center analysis. Radiat Oncol J. 2019;14:162. doi: 10.1186/s13014‐019‐1334‐9 10.1186/s13014-019-1334-9 PMC6724320 31481089

